# Short-term and Long-term Outcomes of Combined Surgical Aortic Valve Replacement and Coronary Artery Bypass Grafting in the Pre-TAVI Era: Insights into Contemporary Treatment Selection for Aortic Stenosis with Coronary Artery Disease

**DOI:** 10.14789/ejmj.JMJ25-0010-OA

**Published:** 2025-06-20

**Authors:** HIROSHI NAKAMURA, KAN KAJIMOTO, TAIRA YAMAMOTO, RYOMA ODA, TAKESHI KINOSHITA, ATSUSHI AMANO, MINORU TABATA

**Affiliations:** 1 Department of Cardiovascular Surgery, Juntendo University, School of Medicine, Tokyo, Japan; 1 Department of Cardiovascular Surgery, Juntendo University, School of Medicine, Tokyo, Japan

**Keywords:** aortic stenosis, aortic valve replacement, concomitant, coronary artery bypass grafting, elderly

## Abstract

**Objectives:**

Transcatheter aortic valve implantation (TAVI) has replaced combined coronary artery bypass grafting (CABG) and surgical aortic valve replacement (SAVR) as the standard treatment for elderly patients with severe coronary artery disease (CAD) and aortic valve stenosis (AS). However, the long-term outcomes of the surgical approaches in the pre-TAVI era need revisiting. This study evaluated the short- and long-term outcomes of combined SAVR and CABG in elderly patients during the pre-TAVI era.

**Materials:**

This retrospective data analysis evaluated patients aged ≥ 70 years who underwent combined SAVR and CABG between 2005 and 2014.

**Methods:**

Short-term outcomes, including in-hospital mortality, stroke, respiratory failure and acute kidney injury, and long-term outcomes such as all-cause mortality, cardiac mortality, and major adverse cardiac and cerebrovascular events (MACCE), were assessed.

**Results:**

Among 123 patients (mean age: 78 years), risk scores confirmed an intermediate risk (EuroSCORE II: 6.1%, JapanSCORE: 7%, JapanSCORE II: 21%), with rates of in-hospital mortality of 3.3%, stroke of 5.7%, and respiratory failure of 22%. Five- and 10-year survival rates were 66.3% and 42.6%, respectively, cardiac mortality rates were 4.8% and 7.2%, respectively, and event-free survival for MACCE were 66.6% and 55.1%, respectively.

**Conclusions:**

The outcomes of SAVR+CABG in the pre-TAVI era demonstrate the efficacy and durability of surgical approaches, with favorable long-term survival even in elderly intermediate risk patients. These findings highlight the continued significance of surgical intervention, particularly for patients requiring treatment for both AS and CAD, and provide a benchmark for assessing current treatment strategies in the TAVI era.

## Introduction

The treatment paradigm for aortic stenosis (AS) has evolved significantly with the introduction and subsequent expansion of the indications of transcatheter aortic valve implantation (TAVI). Initially indicated for patients with a high surgical risk, randomized controlled trials, such as PARTNER 3 and Evolut Low Risk, have extended the indications of TAVI to intermediate- and low-risk populations, with favorable outcomes^1, 2)^.

Simultaneously, aging of the population has increased the prevalence of patients with concomitant AS and coronary artery disease (CAD), many of whom require comprehensive treatment strategies^3)^. Historically, surgical aortic valve replacement (SAVR) combined with coronary artery bypass grafting (CABG) was the standard approach to these patients, particularly in the pre-TAVI era. However, while TAVI + percutaneous coronary intervention (PCI) has become more common due to its limited invasiveness, its long-term outcomes in complex CAD remain uncertain^4)^.

Recent studies have suggested that SAVR + CABG continues to provide excellent outcomes, particularly in patients requiring complete revascularization and AVR, with lower rates of cardiac mortality and major adverse cardiac and cerebrovascular events (MACCE) compared to TAVI + PCI in intermediate-risk patients^5)^.

Given these considerations, it is crucial to revisit the outcomes of combined CABG and SAVR in the pre-TAVI era, particularly in elderly patients. This study aimed to evaluate the short- and long-term outcomes of combined SAVR + CABG in patients aged 70 years and older, focusing on the efficacy and durability of this surgical approach in managing complex cardiovascular diseases.

## Methods

### Ethics statement

All study protocols received approval from the Research Ethics Committee of Juntendo University's Faculty of Medicine on March 6, 2025 (approval no. E24-0492). Given the retrospective study design, the requirement for informed consent was waived. The study was conducted following the ethical principles outlined in the Declaration of Helsinki.

### Study design and inclusion criteria

**Figure 1:**
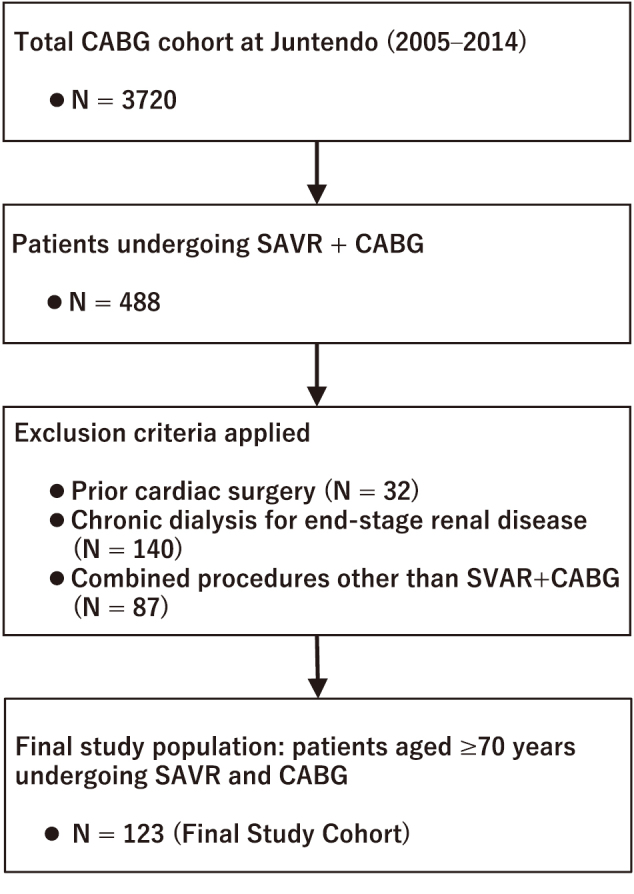
Flow chart of patient selection

This retrospective, observational, single institution analysis included patients aged ≥ 70 years who underwent combined CABG and SAVR between 2005 and 2014. A flow chart of patient selection was shown in Figure 1. All the patients presented with concomitant severe CAD and AS requiring surgical intervention. The exclusion criteria were prior cardiac surgery, chronic dialysis for end-stage renal disease, and concomitant procedures other than CABG and SAVR, such as mitral valve surgery or aortic surgery.

### Patient data and follow-up

Data was collected retrospectively from institutional surgical and clinical databases, and were used for recording patient baseline characteristics, operative details, and postoperative outcomes. Both short-term and long-term outcomes were assessed as study endpoints. Short-term outcomes included in-hospital mortality, stroke, respiratory failure and acute kidney injury (AKI), while long-term outcomes included all-cause mortality, cardiac mortality and MACCE. Long-term outcomes were evaluated via serial follow-up contacts (every five years) with patients or their families, continuing until December 2020.

### Definitions

Postoperative respiratory failure was defined as the need for prolonged (> 48 hours) mechanical ventilation or pleural effusion requiring percutaneous drainage. Postoperative stroke was defined as a new stroke diagnosed via MRI or computed tomography. AKI was defined as a greater than 50% increase in serum creatinine from baseline. The severity of AS was categorized according to the ACC/AHA Guidelines for the Management of Valvular Heart Disease^4)^. Moderate AS was defined as an aortic valve orifice area of 1.0-1.5 cm^2^, and severe AS as an orifice area of ≤ 1.0 cm^2^, as estimated using echocardiographic aortic jet velocity. Cardiac death was defined as death resulting from myocardial infarction, congestive heart failure, arrhythmia, or sudden cardiac death. MACCE was defined as a composite of all-cause death, myocardial infarction and stroke.

### Statistical analysis

All data were analyzed using descriptive statistics. Continuous variables are presented as the mean ± standard deviation, while categorical variables are summarized as frequencies and percentages. Long-term survival was evaluated using Kaplan-Meier survival curves, which provided a graphical representation of survival probabilities over time. As this study focused on describing outcomes in the pre-TAVI cohort, no formal statistical comparisons between groups were performed. Statistical analyses were conducted using SPSS Statistics software (version 16.0; IBM, Armonk, NY, USA).

## Results

### Baseline characteristics

A total of 123 patients (mean age: 78 ± 5 years) who underwent combined SVAR and CABG in the pre-TAVI era were included in this study (Table 1). The majority of patients had a history of diabetes mellitus. Hypertension was prevalent in 84% (n = 103) of the patients, and 43% (n = 53) had a history of smoking. Renal function, assessed using the estimated glomerular filtration rate (eGFR), indicated a mean value of 61 ± 21 mL/min/1.73 m^2^. Baseline lipid levels, including mean total cholesterol (174 ± 34 mg/dL) and low-density lipoprotein (LDL) cholesterol (103 ± 32 mg/dL), were within standard ranges for the population. Additionally, the mean aortic valve area (AVA) was 0.77 ± 0.24 cm^2^, with a mean peak aortic valve pressure gradient of 48 ± 24 mmHg, indicating severe AS in most cases. Left ventricular ejection fraction (LVEF) was preserved in the majority of patients, with a mean value of 62 ± 13%. The estimated mortality rates were 6.1% for EuroSCORE II, 7% for JapanSCORE and 21% for JapanSCORE.

**Table 1:** Baseline characteristics of the study subjects

Variables	Patients (n = 123)
Age (years), mean ± SD	78 ± 5
Male, n (%)	61 (50%)
Diabetes mellitus, n (%)	45 (37%)
Hypertension, n (%)	103 (84%)
Smoking history, n (%)	53 (43%)
Family history, n (%)	31 (25%)
Chronic kidney disease, n (%)	7 (5.7%)
History of stroke, n (%)	20 (16%)
Peripheral artery disease, n (%)	31 (25%)
Pulmonary disease, n (%)	24 (20%)
Emergency surgery, n (%)	1 (0.8%)
HbA1c (%), mean ± SD	5.8 ± 0.8
Fasting blood glucose (mg/dL), mean ± SD	106 ± 31
Total cholesterol (mg/dL), mean ± SD	174 ± 34
Triglycerides (mg/dL), mean ± SD	101 ± 43
LDL cholesterol (mg/dL), mean ± SD	103 ± 32
HDL cholesterol (mg/dL), mean ± SD	51 ± 14
eGFR (mL/min/1.73 m^2^), mean ± D	61 ± 21
BNP (pg/mL), mean ± SD	371 ± 536
AV mean pressure gradient (mmHg), mean ± SD	48 ± 24
Aortic valve area (cm^2^), mean ± SD	0.77 ± 0.24
Aortic peak velocity (m/s), mean ± SD	4.1 ± 1.0
Left ventricular ejection fraction (%), mean ± SD	62 ± 13
EuroSCORE II, mean ± SD	6.1 ± 8.1
JapanSCORE, mean ± SD	7 ± 11
JapanSCORE II, mean ± SD	21 ± 15

SD, standard deviation; HbA1c, hemoglobin A1c; LDL, low-density lipoprotein; HDL, high-density lipoprotein; eGFR, estimated glomerular filtration rate; BNP, brain natriuretic peptide.

### Operative details

Intraoperative parameters in this study subjects were shown in Table 2. The mean number of distal anastomoses was 2.15 ± 1.2 per patient. Bilateral internal thoracic arteries (ITAs) (left ITA (LITA) and right ITA (RITA)) were utilized in 31% (n = 38) of patients, while the LITA alone was used in 40% (n = 49) of cases. Radial artery grafts were employed in 8.1% (n = 10) of patients, and saphenous vein grafts (SVG) in 66% (n = 81). The mean cardiopulmonary bypass time was 142 ± 34 minutes, and the mean aortic cross-clamp time was 96 ± 28 minutes. Regarding the valve procedure, all patients underwent SAVR with bioprosthetic valve replacement. The mean prosthetic valve size was 23.1 ± 2.1 mm. No intraoperative conversions to other surgical techniques or emergency procedures were required.

**Table 2:** Intraoperative and postoperative parameters in the study subjects

Variables	Patients
Operative parameters	
Number of distal anastomoses, mean ± SD	2.15 ± 1.2
Use of LITA, n (%)	49 (40%)
Use of RITA, n (%)	38 (31%)
Use of RA, n (%)	10 (8.1%)
Postoperative parameters	
ICU stay (days), mean ± SD	4.8 ± 8.9
Hospital stay (days), mean ± SD	20 ± 21
Postoperative complications	
In-hospital death, n (%)	4 (3.3%)
Stroke, n (%)	7 (5.7%)
Respiratory failure, n (%)	27 (22%)
Acute kidney injury, n (%)	17 (14%)

LITA, left internal thoracic artery; RITA, right internal thoracic artery; RA, radial artery.

### Short-term outcomes

Short-term outcomes following combined CABG and SAVR demonstrated favorable results. The observed in-hospital mortality rate was 3.3% (n = 4), and postoperative stroke occurred in 5.7% (n = 7) of patients. Respiratory failure requiring prolonged mechanical ventilation or drainage of pleural effusion was observed in 22% (n = 27) of patients. AKI occurred in 14% (n = 17) of patients. None of the patients required reoperation for postoperative bleeding. The mean duration of intensive care unit (ICU) stay was 4.8 ± 8.9 days, and the mean total duration of hospitalization was 20 ± 21 days.

### Long-term outcomes

**Figure 2:**
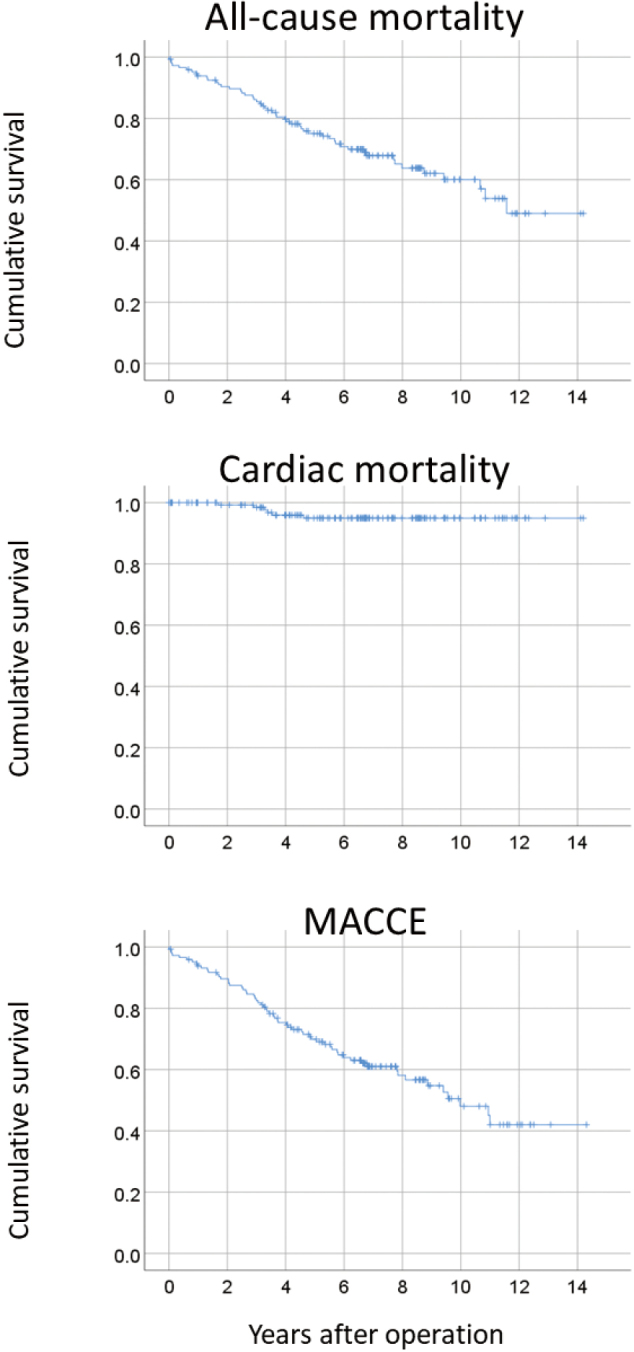
Cumulative event-free survival curves

The mean follow-up periods were 5.9 years. The results of cumulative survival analyses are shown in Figure 2. Kaplan-Meier survival analysis of all- cause mortality showed cumulative survival rates of 90.9% (standard error: 2.2%) at 1 year, 66.3% (standard error: 3.7%) at 5 years, and 42.6% (standard error: 6.4%) at 10 years. For cardiac mortality, cumulative survival rates were 98.8% (standard error: 0.9%) at 1 year, 95.2% (standard error: 1.8%) at 5 years, and 92.8% (standard error: 2.2%) at 10 years. The cumulative event-free survival rate for MACCE was 90.2% (standard error: 2.3%) at 1 year, 66.6% (standard error: 3.9%) at 5 years, and 55.1% (standard error: 4.0%) at 10 years.

## Discussion

This study demonstrates that combined SAVR and CABG achieved favorable short- and long-term outcomes in an elderly and intermediate-risk Japanese cohort during the pre-TAVI era. Despite the absence of less invasive treatments such as TAVI, the observed in-hospital mortality rate was 3.3%, which was markedly lower than that predicted by risk scores (EuroSCORE II: 6.1%, JapanSCORE: 7%, and JapanSCORE II: 21%). In addition to the lower-than-expected in-hospital mortality, other short-term outcomes including incidence of postoperative stroke (5.7%), respiratory failure rate (22%) and acute kidney injury (14%) in our cohort were consistent with or better than previously reported data for SAVR + CABG in elderly patients^5, 6)^. Furthermore, the 5-year survival rate of 66.3% and 10-year survival rate of 42.6% reinforce the safety and efficacy of this surgical approach, particularly for intermediate-risk elderly patients requiring complete revascularization.

Despite the expanding indications of TAVI + PCI, its long-term outcomes remain unclear. While TAVI + PCI offers a less invasive treatment strategy, concerns persist regarding its durability, particularly for intermediate-risk patients with severe CAD. In contrast, this study highlights the favorable short- and long-term outcomes of SAVR + CABG in an elderly, intermediate-risk Japanese cohort during the pre-TAVI era.

Observational data from the OBSERVANT study demonstrated 3-year survival rates of 74.2% for SAVR + CABG versus 65.0% for TAVI + PCI, with no significant differences in MACCE^5)^. On the other hand, reports on TAVI + PCI outcomes revealed potential advantages in selected patients, especially in patients with less complex CAD, represented by lower SYNTAX scores. For instance, the SURTAVI trial reported 2-year mortality rates of 16.0% for TAVI + PCI compared to 14.0% for SAVR + CABG in intermediate-risk patients^6)^. However, studies have also suggested that TAVI + PCI is more likely to result in incomplete revascularization. Fortmeier et al. reported higher residual SYNTAX scores for TAVI + PCI compared to SAVR + CABG (5.0 vs. 0.0, p = 0.03), reflecting the limitations of PCI in achieving complete revascularization^7)^. A meta- analysis by Guo et al. further revealed the limitations of TAVI + PCI, showing higher rates of coronary reintervention and lower long-term survival compared to SAVR + CABG^8)^.

The meta-analysis by Sá et al. further supports these findings, demonstrating that complete surgical revascularization with SAVR + CABG resulted in superior long-term survival and lower adverse events than TAVI + PCI, particularly in patients with severe CAD^9)^. Additionally, Griese et al. observed higher early mortality rates (15% vs. 5%) and late mortality rates in TAVI + PCI compared to SAVR + CABG^10)^. Ullah et al. revealed trends indicating higher rehospitalization rates and adverse outcomes in patients treated with TAVI + PCI compared to SAVR + CABG, based on a national database^11)^. These findings suggest that incomplete revascularization and residual coronary lesions in TAVI + PCI might result in worse long-term outcomes.

Based on these findings and the results of our study, SAVR + CABG remains a reasonable treatment option for elderly and intermediate-risk patients with AS and CAD. While TAVI + PCI might be appropriate for patients with lower SYNTAX scores and less complex CAD, the surgical approach offers better outcomes in patients requiring complete revascularization. This study establishes a benchmark for evaluating the long-term efficacy of TAVI + PCI for future studies that might report on the 5- and 10-year outcomes for TAVI + PCI.

Finally, the 10-year survival rate of 42.6% observed in this study is notable, given the average life expectancy of 9.09 years for 78-year-old Japanese men and 12.99 years for women^12)^. The findings of this study suggest that SAVR + CABG not only offers survival comparable to life expectancy, but might also exceed expectations in terms of quality- adjusted life years.

## Limitations

This study has several limitations. The retrospective design and absence of direct comparisons with PCI + TAVI reduce its applicability to current clinical practice. Furthermore, this study is based on descriptive statistics without a control group, it cannot make direct comparisons to other treatment strategies. Additionally, multivariable analyses were not conducted, preventing the identification of specific predictors of adverse outcomes. Detailed data on the specific causes of non-cardiac deaths and SYNTAX score were not available in our records.

## Conclusion

This study demonstrates that SAVR + CABG provided favorable short- and long-term outcomes in an elderly and intermediate-risk cohort of Japanese patients in the pre-TAVI era. The lower-than-expected mortality rates and survival comparable to predicted life expectancy reinforce the continued relevance of surgical approaches, particularly for patients requiring complete revascularization and definitive valve replacement. Our study helps establish a benchmark for the future assessment of TAVI + PCI outcomes as longer-term data becomes available in the future.

## Author contributions

HN analyzed and interpreted patient data and was a major contributor in writing the manuscript. KK contributed to the design of the work and revised the manuscript critically. YT was a major contributor in the acquisition of data for the work. RO contributed to ethical approvement. All authors read and approved of the final manuscript.

## Conflicts of interest statement

The authors declare that there are no conflicts of interest.
